# Supplementary material for: Improving the safety of human pluripotent stem cell therapies using genome-edited orthogonal safeguards
Source: Nat Commun. 2020 Jun 1;11:2713. doi: 10.1038/s41467-020-16455-7 (PMC7264334; doi:10.1038/s41467-020-16455-7)
Supplement: Supplementary file 1 — Supplementary Information [file 41467_2020_16455_MOESM1_ESM.pdf]

Improving the safety of human pluripotent stem cell therapies using genome-edited orthogonal safeguards

Martin & Fowler et al.

Supplementary Figure 1

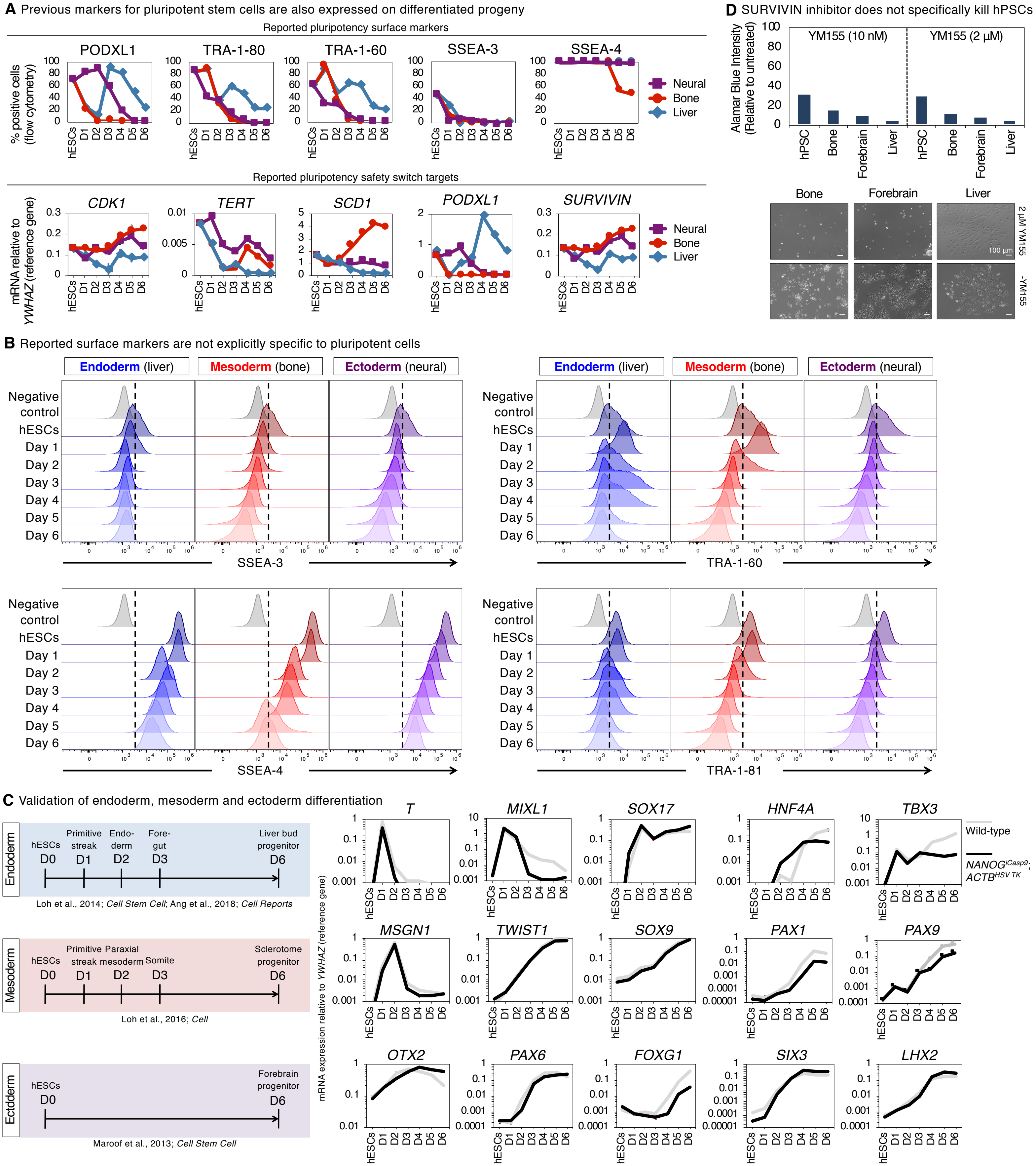

Supplementary Figure 1: Marker expression during hPSC differentiation and evaluation of past safety switches

A) Flow cytometry analysis of widely-used pluripotency surface markers SSEA-3, SSEA-4, TRA-1-60, TRA-1-81 and PODXL1[1,2] before and during differentiation into endodermal, mesodermal and ectodermal lineages for 1-6 days. Positive gates were set based on unstained negative controls (*top*). Gene expression analysis of previously reported safety system genes *CDK1, TERT, SCD1, PODXL1* and *SURVIVIN* before and during differentiation into endodermal, mesodermal and ectodermal lineages. Expression of lineage markers is depicted normalized to the reference gene *YWHAZ* (i.e., *YWHAZ* = 1.0) (*bottom*). Error bars = standard error.

B) Widely-used pluripotency surface markers such as SSEA-3, SSEA-4, TRA-1-60, TRA-1-81 and PODXL1[1,2] were not exclusive to pluripotent cells. Flow cytometry analysis of undifferentiated hESCs and upon 1-6 days of differentiation into endodermal, mesodermal or ectodermal lineages revealed that these surface markers were expressed in both undifferentiated and differentiated cell-types. Unstained hESCs (top row; grey shading) were used as a negative control to set positive gates (dotted vertical lines).

C) Endodermal[3,4], mesodermal[5] and ectodermal[6] differentiation protocols used in this study were validated by assessing expression of lineage-specific markers during differentiation into each of these respective cell-types. qPCR was performed on wild-type H9 hESCs (grey line) or $NANOG^{iCasp9\text{-}YFP}$;$ACTB^{TK\text{-}mPlum}$ hPSCs (black line) in the undifferentiated state or upon 1-6 days of differentiation into endodermal, mesodermal or ectodermal lineages. This analysis also revealed that genetic targeting of the *NANOG* and *ACTB* loci did not significantly perturb differentiation into these 3 cell-types. Expression of lineage markers is depicted normalized to the reference gene *YWHAZ* (i.e., *YWHAZ* = 1.0), with y-axis expression values depicted in log_{10}. Error bars = standard error.

D) H9 undifferentiated hPSCs and hPSC-derived neural, liver, and sclerotome cells (generated after 6 days of differentiation) were treated for 24 hours with YM155 (either 10 nM or 2 µM), a small molecule SURVIVIN inhibitor[7,8], and cell viability was assessed using alamar blue.

Supplementary Figure 2

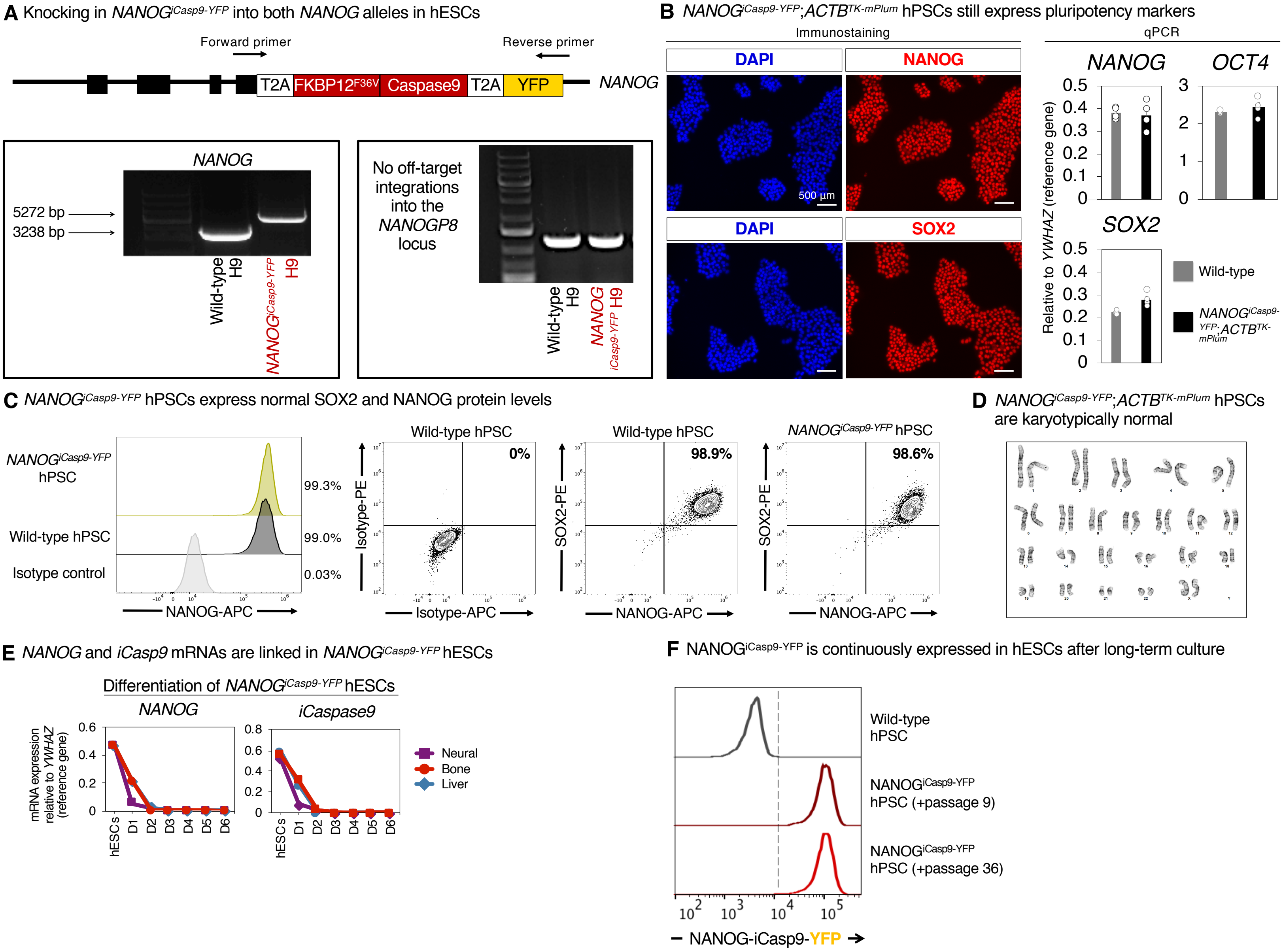

Supplementary Figure 2: Construction of the *NANOG*$^{iCasp9\text{-}YFP}$ safety switch

A) Schema of targeted *NANOG*$^{iCasp9\text{-}YFP}$ allele (see **Fig. 2c**), with forward and reverse primers used for genotyping indicated (*top*). Genomic PCR revealed biallelic targeting of the *NANOG* locus (*left*), with no off-target integrations into the related *NANOGP8* locus (*right inset*).

B) *NANOG*$^{iCasp9\text{-}YFP}$;*ACTB*$^{TK\text{-}mPlum}$*NANOG*$^{iCasp9\text{-}YFP}$ hPSCs were still pluripotent. They uniformly expressed SOX2 and NANOG at both the protein level (immunostaining; *left*) and mRNA level (qPCR; *right*). For immunostaining, DAPI was used for nuclear counterstaining. For qPCR, wild-type hPSCs were used as a positive control, and expression of marker genes is depicted normalized to the reference gene *YWHAZ* (i.e., *YWHAZ* = 1.0). Error bars = standard error.

C) In *NANOG*$^{iCasp9\text{-}YFP}$ hPSCs, SOX2 and NANOG proteins were still expressed at the normal levels found in wild-type hESCs, as shown by intracellular flow cytometry. Isotype controls (grey) were used to set positive gates.

D) *NANOG*$^{iCasp9\text{-}YFP}$;*ACTB*$^{TK\text{-}mPlum}$ hPSCs were karyotypically normal (9 passages after initial *NANOG*$^{iCasp9\text{-}YFP}$ targeting).

E) qPCR of *NANOG*$^{iCasp9\text{-}YFP}$ hPSCs differentiating into endodermal, mesodermal or ectodermal cell-types (differentiation protocols described in **Supplementary Fig.1c**) shows that expression of *iCaspase9 (FKBP-Casp9)* mRNA and endogenous *NANOG* mRNA is similar in both hESCs and differentiated cell-types, consistent with how they are transcriptionally linked in the *NANOG*$^{iCasp9\text{-}YFP}$ allele. Expression of marker genes is depicted normalized to the reference gene *YWHAZ* (i.e., *YWHAZ* = 1.0). Error bars = standard error.

F) Despite short-term (passage 9) or long-term (passage 36) culture, the *NANOG*$^{iCasp9\text{-}YFP}$ allele was constitutively expressed in undifferentiated *NANOG*$^{iCasp9\text{-}YFP}$ hPSCs, as shown by flow cytometry. (Passages refer to the time when the *NANOG*$^{iCasp9\text{-}YFP}$ allele was first introduced into hPSCs.)

Supplementary Figure 3

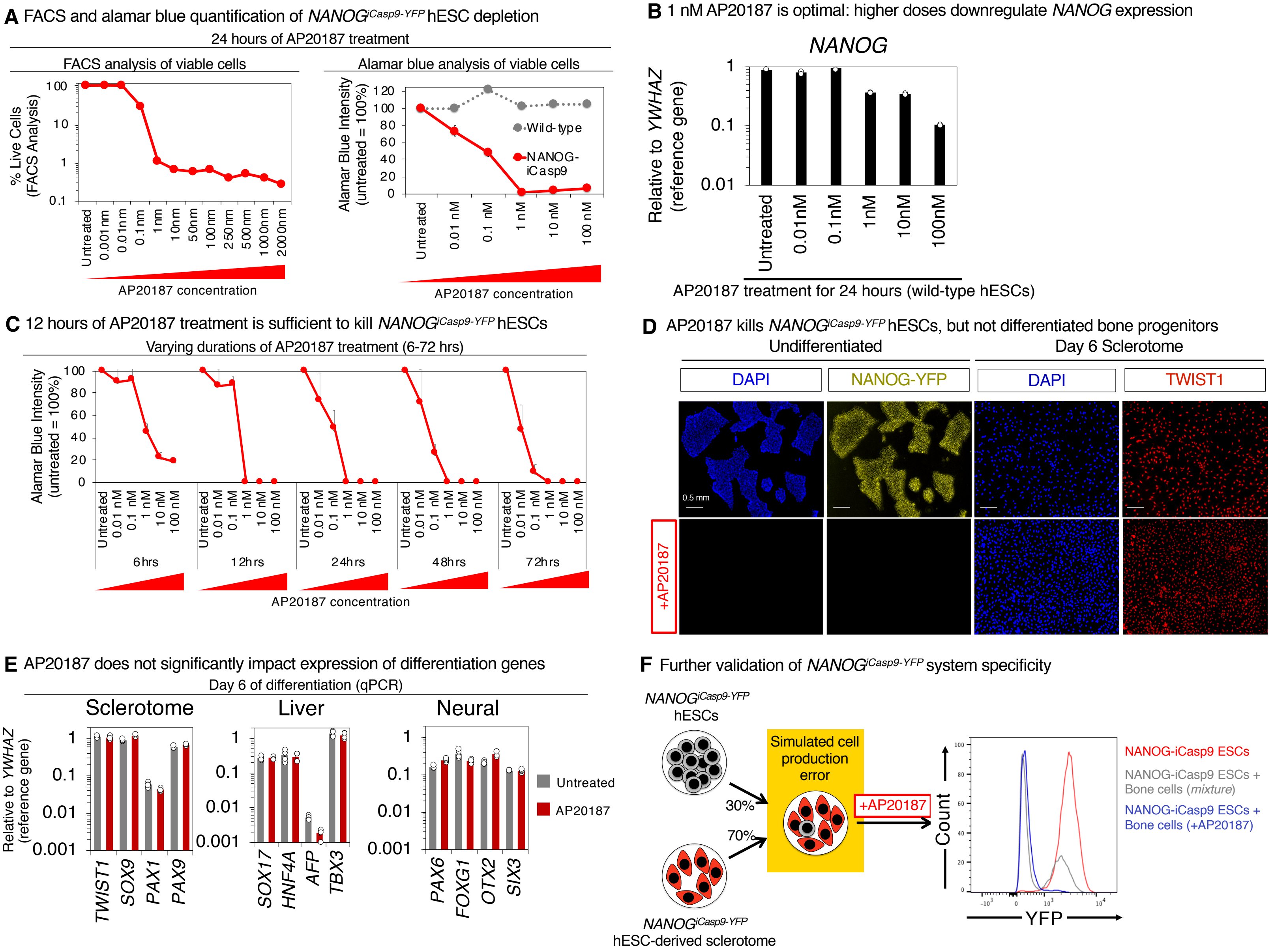

Supplementary Figure 3: Efficacy of the *NANOG*$^{iCasp9\text{-}YFP}$ safety switch

A) Undifferentiated *NANOG*$^{iCasp9\text{-}YFP}$ hPSCs were treated with AP20187 at the indicated concentrations for 24 hours, and then the percentage of viable remaining cells was subsequently analyzed using FACS analysis (*left*) and alamar blue staining (*right*). For the FACS analysis, the percentage of cells shown represents viable cells (i.e., DAPI-negative cells obtained after DAPI staining) that were then gated for YFP+ (i.e., NANOG^{+}) cells. For the alamar blue analysis, wild-type hPSCs were used as a negative control. Error bars = standard error.

B) qPCR indicated that treatment with increasing doses of AP20187 downregulated *NANOG* mRNA expression in undifferentiated hPSCs. AP20187 doses equal to or greater than 100 nM may prevent efficient killing of *NANOG*$^{iCasp9\text{-}YFP}$ hPSCs by considerably downregulating *NANOG*. Gene expression is depicted normalized to the reference gene *YWHAZ* (i.e., *YWHAZ* = 1.0). Error bars = standard error.

C) Undifferentiated *NANOG*$^{iCasp9\text{-}YFP}$ hPSCs were treated with the indicated doses of AP20187 for various lengths of time (6, 12, 24, 48, 72 hours) and then alamar blue assay was performed immediately thereafter to quantify the extent of cell death. This revealed that AP20187-induced cell death occurs within 12 hours of treating *NANOG*$^{iCasp9\text{-}YFP}$ hPSCs with 1 nM of AP20187. Error bars = standard error.

D) Immunofluorescent imaging of *NANOG*$^{iCasp9\text{-}YFP}$ hESCs in the undifferentiated state and after differentiation into day 6 sclerotome as marked by TWIST1 expression, both without and with treatment of cells with 1 nM AP20187 for 24 hours.

E) Transcriptional analysis of differentiated cell-types before and after AP20187 (1 nM) treatment for 24 hours showed that AP20187 treatment did not substantially impact marker gene expression. The following marker genes were assessed in each respective cell-type: sclerotome (bone) progenitors (*TWIST1, SOX9, PAX1, PAX9),* liver progenitors (*SOX17, HNF4A, AFP, TBX3*), and forebrain (neural) progenitors (*PAX6, FOXG1, OTX2, SIX3*). Error bars = standard error.

F) Further validation of *NANOG*$^{iCasp9\text{-}YFP}$ using a simulated mixed cell culture assay. *NANOG*$^{iCasp9\text{-}YFP}$ undifferentiated hPSCs and hPSC-derived derived sclerotome cells were mixed at a 3:7 ratio, respectively. Mixed cells were treated with AP20187, and 24 hours post-treatment, FACS analysis was done to assess remaining *NANOG*$^{iCasp9\text{-}YFP}$ hPSCs in culture.

Supplementary Figure 4

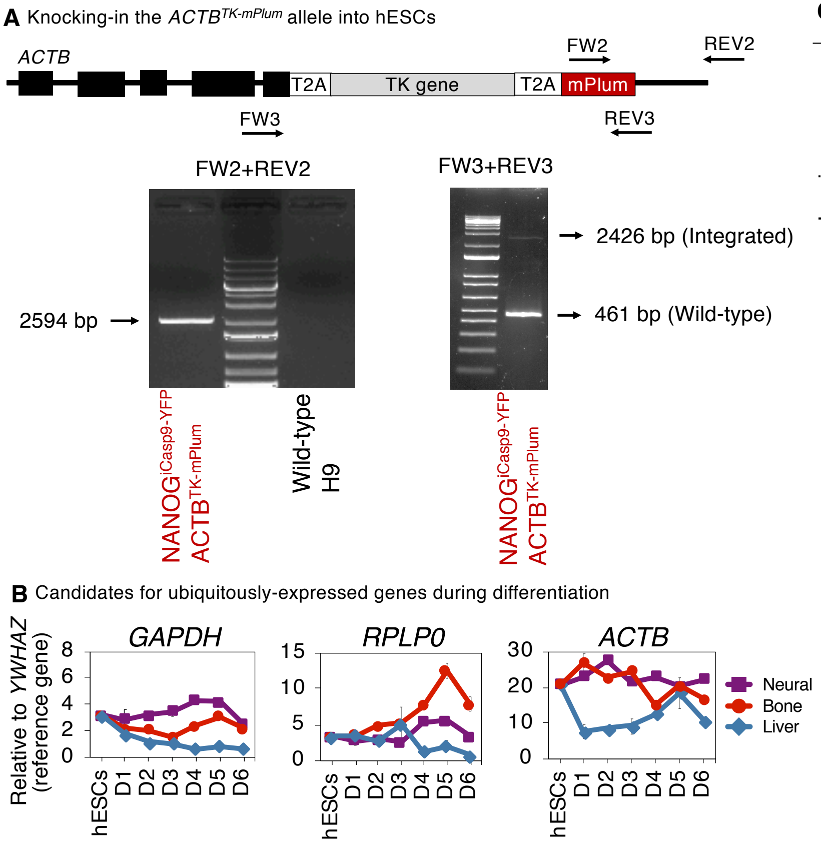

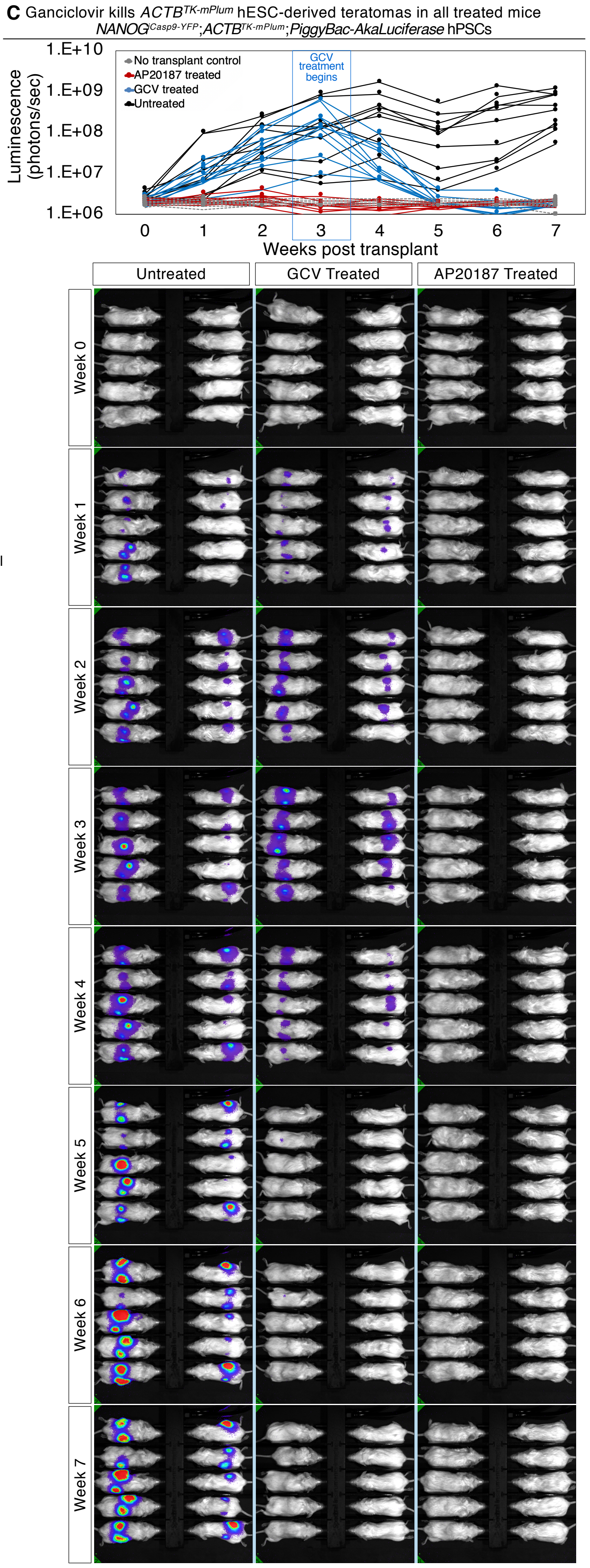

Supplementary Figure 4: Supporting data for *ACTB*$^{TK\text{-}mPlum}$ safety switch

A) Schema of targeted *ACTB*$^{TK\text{-}mPlum}$ allele, with forward and reverse primers used for genotyping indicated. Genomic in-out PCR showing 2594bp band for the C-terminal-end integrated sequence revealed targeting of the *ACTB* locus and PCR confirming mono-allelic integration of *ACTB*$^{TK\text{-}mPlum}$*.*

B) qPCR to assess the expression of *GADPH*, *RPLP0*, *ACTB* mRNAs during neural, bone and liver differentiation revealed that they are all ubiquitously expressed, with *ACTB* showing the highest expression levels. Expression of lineage markers is depicted normalized to the reference gene *YWHAZ* (i.e., *YWHAZ* = 1.0). Error bars = standard error.

C) 10^6 *ACTB*$^{TK\text{-}mPlum}$*;NANOG*$^{iCasp9\text{-}YFP}$ hESCs engineered to express AkaLuciferase were treated with control media or 1 nM AP20187 for 24 hours, and then subcutaneously transplanted into the left and right dorsal flanks of NOD-SCID *Il2rg*$^{-/-}$ mice (10^6 cells per flank. After 3 weeks post-transplant, teratomas formed *in vivo* and ganciclovir was administered daily at 50mg/kg for 4 further weeks. Bioluminescent imaging of mice occurred weekly for 7 weeks. Total flux (photons/sec) was measured for each animal.

Supplementary Figure 5

A Genetic knock-in efficiencies

Locus	Size/Type of Insertion	Editing Efficiency
NANOG	2082 bp (*iCasp9-YFP*)	1.45%
ACTB	1965 bp (*OiCasp9-mPlum*)	11.7%

B *NANOG$^{iCasp9\text{-}YFP}$;ACTB$^{OiCasp9\text{-}mPlum}$* hPSCs are karyotypically normal

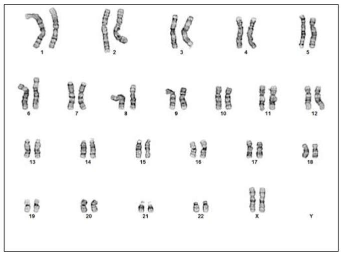

C AP21967 does not activate iCaspase9

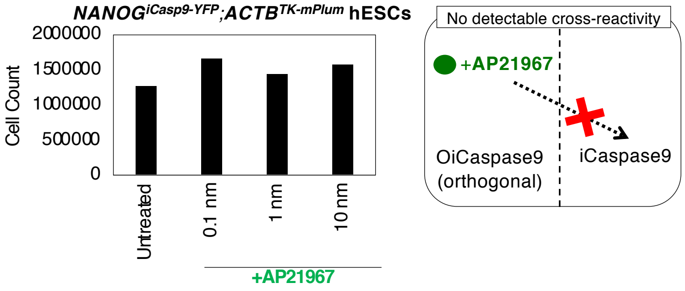

D AP21967 does not activate iCaspase9 in a cell-competition assay

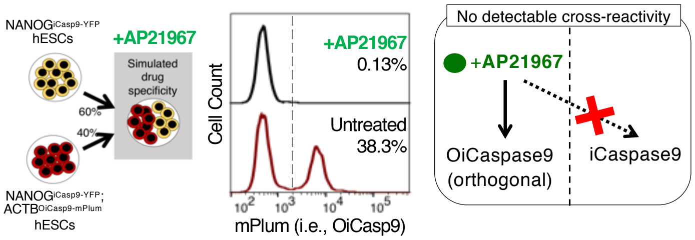

E AP21967 rapidly eliminates *ACTB$^{OiCasp9\text{-}mPlum}$* hESC-derived teratomas in all treated mice

NANOG$^{iCasp9\text{-}YFP}$;ACTB$^{OiCasp9\text{-}mPlum}$;PiggyBac-AkaLuciferase hPSCs

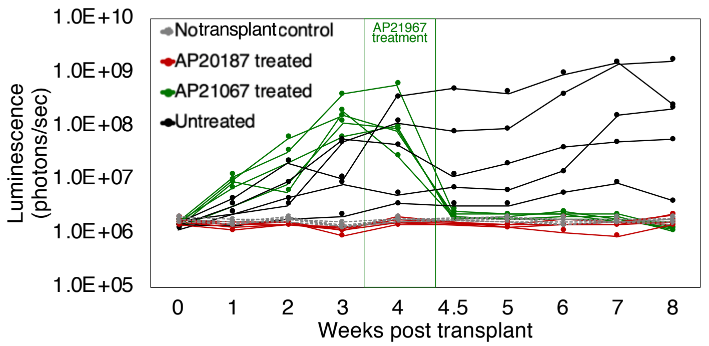

F AP21967 eliminates *ACTB$^{OiCasp9\text{-}mPlum}$* hESC-derived teratomas in all treated mice

NANOG$^{iCasp9\text{-}YFP}$;ACTB$^{OiCasp9\text{-}mPlum}$;PiggyBac-AkaLuciferase hPSCs

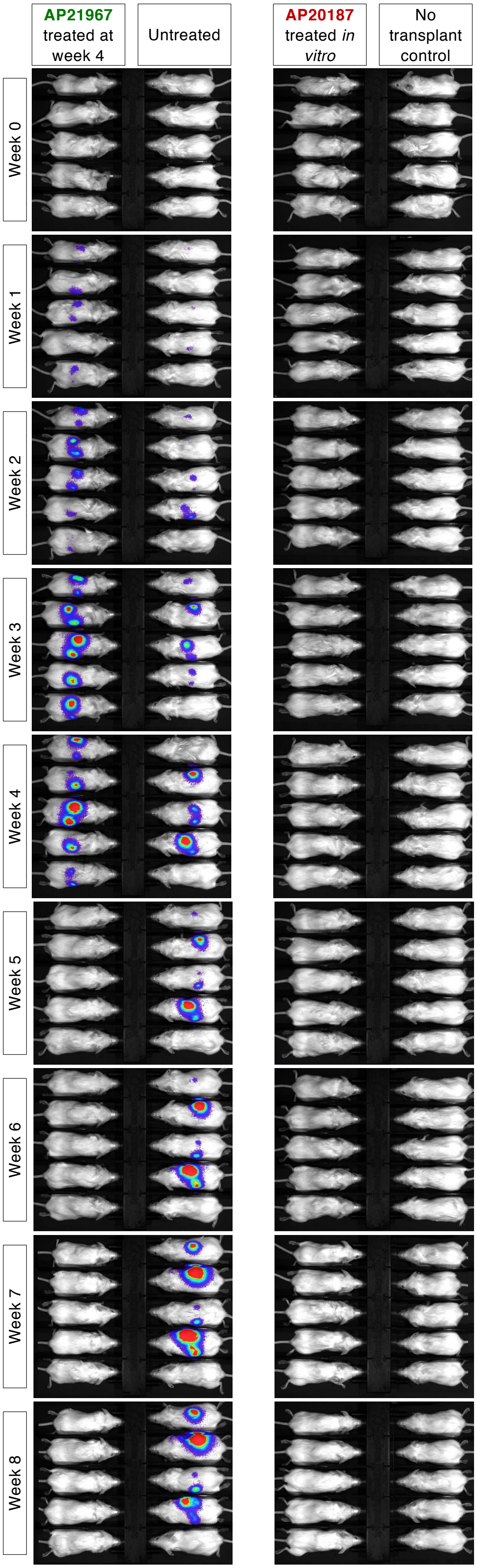

Supplementary Figure 5: Supporting data for *ACTB*$^{OiCasp9\text{-}mPlum}$ safety switch

A) Knock-in efficiencies at the *NANOG* and *ACTB* loci in hPSCs. Targeting efficiencies were quantified by performing flow cytometry of bulk hPSC populations edited through the Cas9 RNP/AAV6 system[9] (prior to single-cell cloning to generate clonal cell lines), and assessing the percentage of cells that expressed the respective fluorescent reporters (YFP, in the case of the *NANOG*$^{iCasp9\text{-}YFP}$ allele or else mPlum, in the case of the *ACTB*$^{OiCasp9\text{-}mPlum}$ allele). Notably, while the Cas9 RNP/AAV6 system has been reported to generate knock-in alleles with 20-60% efficiency in hPSCs at multiple genes that not essential for cellular viability[9], here the targeting efficiencies at the *NANOG* and *ACTB* loci were lower. This is likely because Cas9 inflicts double-strand DNA breaks, which are known to transcriptionally silence nearby genes as part of the DNA damage response[10]. Temporary silencing of *NANOG* and *ACTB* likely led to cell death or differentiation, thus hindering the recovery of successfully-targeted hPSCs.

B) *NANOG*$^{iCasp9\text{-}YFP}$;*ACTB*$^{OiCasp9\text{-}mPlum}$ hESCs were karyotypically normal (36 passages after initial *NANOG*$^{iCasp9\text{-}YFP}$ targeting).

C) To confirm that AP21967 (which activates OiCaspase9) does not kill iCaspase9-expressing cells, *ACTB*$^{TK\text{-}mPlum}$;*NANOG*$^{iCasp9\text{-}YFP}$ hESCs were treated with AP21967 for 24 hours at the indicated doses and then the number of surviving cells was quantified by cell counting.

D) To confirm that AP21967 (which activates OiCaspase9) does not kill iCaspase9-expressing cells, a mixture of 40% *NANOG*$^{iCasp9\text{-}YFP}$ hESCs + 60% doubly-transgenic *ACTB*$^{OiCasp9\text{-}mPlum}$;*NANOG*$^{iCasp9\text{-}YFP}$ hESCs was either left untreated or treated with AP21967 (1 nM) for 24 hours, and the proportion of surviving cells was quantified by flow cytometry.

E) 10^6 *ACTB*$^{OiCasp9\text{-}mPlum}$;*NANOG*$^{iCasp9\text{-}YFP}$ hESCs engineered to express *CAG-AkaLuciferase* were treated with control media or 1 nM AP20187 for 24 hours, and then subcutaneously transplanted into the left and right dorsal flanks of NOD-SCID *Il2rg*$^{-/-}$ mice (10^6 cells per flank). 4 weeks post-transplant, teratomas formed *in vivo* and AP21967 was intraperitoneally administered once at 10 mg/kg. Bioluminescent imaging of mice was conducted weekly for 8 weeks (with the exception of week 4, when imaging was performed again 3 days post-AP21967 administration). Total flux (photons/sec) was measured for each individual animal.

F) Bioluminescent imaging of individual animals shown in **Supplementary Fig.5e**.

Supplementary Table 1: Quantitative PCR primers

Gene Name	Forward	Reverse
YWHAZ	GAGCTGGTTCAGAAGGCCAAAC	CCTTGCTCAGTTACAGACTTCATGCA
SOX2	TGGACAGTTACGCGCACAT	CGAGTAGGACATGCTGTAGGT
OCT4	AGTGAGAGGCAACCTGGAGA	ACACTCGGACCACATCCTTC
NANOG	CATGAGTGTGGATCCAGCTTG	CCTGAATAAGCAGATCCATGG
KLF4	AGCCTAAATGATGGTGCTTGGT	CCTTGTCAAAGTATGCAGCAGT
CDK1	AAACTACAGGTCAAGTGGTAGCC	TCCTGCATAAGCACATCCTGA
TERT	AAA TGC GGC CCC TGT TTC T	CAG TGC GTC TTG AGG AGC A
SCD1	TCTAGCTCCTATACCACCACCA	TCGTCTCCAACTTATCTCCTCC
PODXL1	TCCCAGAATGCAACCCAGAC	GGTGAGTCACTGGATACACCAA
SURVIVIN/BIRC5	AGGACCACCGCATCTCTACAT	AAGTCTGGCTCGTTCTCAGTG
BRACHYURY	TGCTTCCCTGAGACCCAGTT	GATCACTTCTTTCCTTTGCATCAA G
MIXL1	GGTACCCCGACATCCACTTG	TAATCTCCGGCCTAGCCAAA
SOX17	CGCACGGAATTTGAACAGTA	GGATCAGGGACCTGTCACAC
HNF4A	TCATGCAGGTGTGTGAGT CCA T	AGTCATTGC CTA GGA GCA GCA C
TBX3	TTA CCA AGT CGG GAA GGC GAA T	CAT CCT CTT TGG CAT TTC GGG G
MSGN1	CGGAATTACCTGCCACCTGT	GGTCTGTGAGTTCCCCGATG
TWIST1	CTGCAGCACCGGCACCGTTT	CCCAACGGCTGGACGCACAC
SOX9	CGTCAACGGCTCCAGCAAGAAC AA	GCCGCTTCTCGCTCTCGTTCAGAAGT
PAX1	CGCTATGGAGCAGACGTATGGC GA	AATGCGCAAGCGGATGGCGTTG
PAX9	TGGTTATGTTGCTGGACATGGG TG	GGAAGCCGTGACAGAATGACTAC CT
OTX2	GGAAGCACTGTTTGCCAAGACC	CTGTTGTTGGCGGCACTTAGCT
PAX6	GCAGATGCAAAAGTCCAGGTG	CAGGTTGCGAAGAACTCTGTTT
FOXG1	CCG CAC CCG TCA ATG ACT T	CCG TCG TAA AAC TTG GCA AAG
SIX3	CTGCCCACCCTCAACTTCTC	GCAGGATCGACTCGTGTTTGT
LHX2	TCGGGACTTGGTTTATCACCT	GCAAGCGGCAGTAGACCAG
iCASPASE9	CCAGATGAGTGTGGGTCAGA	TGCTCAGGATGTAAGCCAAA
GAPDH	GGAGCGAGATCCCTCCAAAAT	GGCTGTTGTCATACTTCTCATGG
RPLP0	AGCCCAGAACACTGGTCTC	ACTCAGGATTTCAATGGTGCC
ACTB	AGAGCTACGAGCTGCCTGAC	AGCACTGTGTTGGCGTACAG

Supplementary Table 2: **Flow cytometry antibodies**

Antibody	Fluorophore	Clone	Dilution	Catalog #
SSEA-3	Alexa Fluor 647	MC-631	1:25	BioLegend 330307
SSEA-4	Alexa Fluor 647	MC-813-70	1:25	BioLegend 330407
TRA-1-81	APC	TRA-1-81	1:25	Stem Cell Technologies 60065AZ.1

TRA-1-60	Alexa Fluor 647	TRA-1-60R	1:25	BioLegend 330605
PODXL1	APC	222328	1:25	R&D Systems FAB1658A
SOX2	PE	245610	1:5	BD 560291
NANOG	APC	REA314	1:10	Miltenyi Biotec 130-120-704

Supplementary Table 3: Immunostaining antibodies and other reagents

**Antibody**	**Dilution**	**Catalog #**
Anti-human TWIST1	1:200	RRID:AB_883292
Anti-human NANOG	1:200	RRID: AB_10559205
Anti-human SOX2	1:200	RRID:AB_2195767
Cy™5 AffiniPure Donkey Anti-Rabbit IgG (H+L)	1:500	RRID: AB_2340607
DAPI	1:5000	RRID: AB_2629482
Hoescht live stain	1:2000	Invitrogen™ Cat# H3569

Supplementary Table 4: **Genotyping primers for genetically-edited hPSCs**

**Primer**	**Sequence**	**Notes**	**Gene Targeted**
FW1	CCACCATTATAGATCTCT	NANOG specific	NANOG
REV1	TGTCATTACGATGCAGCAAA	NANOG specific	NANOG
FW2	AGTTCATGCGCTTCAAGGAG	Binds to mPlum	ACTB
REV2	TGAATGGGGGTTGAATGATTA	ACTB specific	ACTB
FW3	CTCAGATCATTGCTCCTCC	ACTB specific	ACTB
REV3	AGAAGTGGGGTGGCTTTTAG	ACTB specific	ACTB
FW4	GCACATCTTGCCAGGATTTTA	NANOGP8 specific	NANOGP8
REV4	TCCTATGAAGGATGGGAGGA	NANOGP8 specific	NANOGP8

SUPPLEMENTARY REFERENCES

1 Draper, J. S., Pigott, C., Thomson, J. A. & Andrews, P. W. Surface antigens of human embryonic stem cells: changes upon differentiation in culture*. *Journal of anatomy* **200**, 249-258, doi:10.1046/j.1469-7580.2002.00030.x (2002).

2 Choo, A. B. *et al.* Selection against undifferentiated human embryonic stem cells by a cytotoxic antibody recognizing podocalyxin-like protein-1. *Stem Cells* **26**, 1454-1463, doi:10.1634/stemcells.2007-0576 (2008).

3 Ang, L. *et al.* A roadmap for human liver differentiation from pluripotent stem cells. *Cell Reports* **22**, 2190-2205 (2018).

4 Loh, K. M. *et al.* Efficient Endoderm Induction from Human Pluripotent Stem Cells by Logically Directing Signals Controlling Lineage Bifurcations. *Cell Stem Cell* **14**, 237-252 (2014).

5 Loh, K. M. *et al.* Mapping the Pairwise Choices Leading from Pluripotency to Human Bone, Heart, and Other Mesoderm Cell Types. *Cell* **166**, 451-467, doi:10.1016/j.cell.2016.06.011 (2016).

6 Maroof, A. M. *et al.* Directed differentiation and functional maturation of cortical interneurons from human embryonic stem cells. *Cell Stem Cell* **12**, 559-572, doi:10.1016/j.stem.2013.04.008 (2013).

7 Bedel, A. *et al.* Preventing Pluripotent Cell Teratoma in Regenerative Medicine Applied to Hematology Disorders. *Stem Cells Translational Medicine* **6**, 382-393, doi:10.5966/sctm.2016-0201 (2017).

8 Lee, M.-O. *et al.* Inhibition of pluripotent stem cell-derived teratoma formation by small molecules. *Proceedings of the National Academy of Sciences of the United States of America* **110**, E3281-3290, doi:10.1073/pnas.1303669110 (2013).

9 Martin, R. M. *et al.* Highly Efficient and Marker-free Genome Editing of Human Pluripotent Stem Cells by CRISPR-Cas9 RNP and AAV6 Donor-Mediated Homologous Recombination. *Cell Stem Cell* **24**, 821-828.e825, doi:10.1016/j.stem.2019.04.001 (2019).

10 Caron, P., van der Linden, J. & van Attikum, H. Bon voyage: A transcriptional journey around DNA breaks. *DNA Repair* **82**, 102686, doi:10.1016/j.dnarep.2019.102686 (2019).
