# Supplementary material for: Improving the safety of human pluripotent stem cell therapies using genome-edited orthogonal safeguards
Source: Nat Commun. 2020 Jun 1;11:2713. doi: 10.1038/s41467-020-16455-7 (PMC7264334; doi:10.1038/s41467-020-16455-7)
Supplement: Supplementary file 7 — Description of Additional Supplementary Files [file 41467_2020_16455_MOESM7_ESM.pdf]

Title: Supplementary Data 1:
Description: Sequence of NANOG-iCasp9-YFP construct

Title: Supplementary Data 2:
Description: Sequence of ACTB-TK-mPlum construct

Title: Supplementary Data 3:
Description: Sequence of ACTB-OiCasp9-mPlum construct

Title: Supplementary Data 4:
Description: Sequence of homologous recombination donor vector backbone
